# Supplementary material for: Concept design of hybrid-actuated lower limb exoskeleton to reduce the metabolic cost of walking with heavy loads
Source: PLoS One. 2023 May 15;18(5):e0282800. doi: 10.1371/journal.pone.0282800 (PMC10184947; doi:10.1371/journal.pone.0282800)
Supplement: Attachment — Submitted filename: (a)Response to Reviewers.docx [file pone.0282800.s001.docx]

PONE-D-22-32591

Concept Design of Hybrid-Actuated Lower Limb Exoskeleton to Reduce the Metabolic Cost of Walking with Heavy Loads

PLoS ONE

Dear editors and reviewers:

Thank you very much for your time and effort that you have put into reviewing our manuscript. I really appreciate all your comments and suggestions! The manuscript has been carefully revised again according to the comments of the reviewers. Besides, we have revised various types of papers to make them more suitable for PLoS ONE's style requirements and replaced the original blurry pictures with clearer pictures after enhancement. Below are our paper review responses. Below you will find our point-by-point responses to the comments and questions of the reviewers.

**We upload the following files to the website. They are (a) our point-by-point response to the comments (below) (response to reviewers), (b) a revised manuscript with revision marks, (c) a clean updated manuscript without highlights and (d)** **a folder containing all pictures of the manuscript.**

Yours sincerely,

No. 516, Yangpu District military road, Shanghai, China

Corresponding author:

Name: Qiaoling Meng

E-mail: [qiaoling_meng@126.com](mailto:qiaoling_meng@126.com)

[Paper Review Responses]

**Reviewer #1:** This paper proposed a design method for a hybrid-actuated lower limb exoskeleton to reduce the metabolic cost of walking. Three human-machine coupling models with passive springs were simulated and analyzed based on the method of simulation muscle driving. Then, this paper also proposed a control strategy based on the metabolic cost models for hybrid-actuated exoskeletons. The simulated experiments showed that the average metabolic cost decreased by 7.2% with both spring and motor. The work is interesting. The proposed method provides a new solution to improve lower limb exoskeletons.
Overall, the paper is well organized and its presentation is good. However, some minor issues still need to be improved.

**Comments:**

1. The experiment results could be added in the abstract to obtain complete information for the readers.

**Responses:**

Thank you very much for your careful comments. We have added the experimental results in the abstract of the article. Please check out the file (b) that the specific changes with highlight.

**Comments**
2. The captions of figures in this paper are not very clear to the readers. The reviewer suggests that the authors could provide more information about the figures. For instance, Figure 2 (a),(b), and (c) meanings could be added in the caption.
**Responses:**

Thank you very much for your careful comments. We have revised and supplemented the title of all the figures in the article. Please check out the file (b) that the specific changes of the figures title are highlighted.

**Reviewer #2:** This paper proposes the conceptual design method for a hybrid-actuated lower limb exoskeleton based on energy consumption simulation. The work is interesting. The content is meaningful. Minor concerns includes:

**Comments:**
(1) The quality of figures 1-2 should be improved.

**Responses:**

Thank you very much for your careful comments. We have optimized the definition of Figure 1 and Figure 2. Please check ‘Fig 1.tif’ and ‘Fig 2.tif’ in folder (d).

**Comments:**
(2) The abscissa of figure 5(a), 6 and 10(a) should be gait cycle instead of time.

**Responses:**

Thank you very much for your careful comments. We modify the abscissa in Figure 5 (a), Figure 6 and Figure 10 (a) to the gait period. Please check ‘Fig 5.tif’, ‘Fig 6.tif’and ‘Fig 10.tif’ in folder (d).
